# M protein ectodomain-specific immunity restrains SARS-CoV-2 variants replication

**DOI:** 10.3389/fimmu.2024.1450114

**Published:** 2024-10-02

**Authors:** Yibo Tang, Kaiming Tang, Yunqi Hu, Zi-Wei Ye, Wanyu Luo, Cuiting Luo, Hehe Cao, Ran Wang, Xinyu Yue, Dejian Liu, Cuicui Liu, Xingyi Ge, Tianlong Liu, Yaoqing Chen, Shuofeng Yuan, Lei Deng

**Affiliations:** ^1^ Hunan Provincial Key Laboratory of Medical Virology, College of Biology, Hunan University, Changsha, China; ^2^ State Key Laboratory of Emerging Infectious Diseases, Carol Yu Centre for Infection, Department of Microbiology, Li Ka Shing Faculty of Medicine, The University of Hong Kong, Hong Kong, Hong Kong SAR, China; ^3^ School of Public Health, Sun Yat-sen University, Shenzhen, China; ^4^ Laboratory of Infection and Virology, Beijing Pediatric Research Institute, Beijing Children’s Hospital, Capital Medical University, National Center for Children’s Health, Beijing, China; ^5^ National Key Laboratory of Veterinary Public Health and Safety, College of Veterinary Medicine, China Agricultural University, Beijing, China; ^6^ National Medical Products Administration Key Laboratory for Quality Monitoring and Evaluation of Vaccines and Biological Products, Sun Yat-sen University, Guangzhou, China; ^7^ Department of Clinical Microbiology and Infection Control, The University of Hong Kong-Shenzhen Hospital, Shenzhen, China; ^8^ Research and Development Department, Beijing Weimiao Biotechnology Co. Ltd., Beijing, China

**Keywords:** SARS-CoV-2, membrane protein, cross-inhibition, serum neutralizing activity, antibody-dependent cellular cytotoxicity

## Abstract

**Introduction:**

The frequent occurrence of mutations in the SARS-CoV-2 Spike (S) protein, with up to dozens of mutations, poses a severe threat to the current efficacy of authorized COVID-19 vaccines. Membrane (M) protein, which is the most abundant viral structural protein, exhibits a high level of amino acid sequence conservation. M protein ectodomain could be recognized by specific antibodies; however, the extent to which it is immunogenic and provides protection remains unclear.

**Methods:**

We designed and synthesized multiple peptides derived from coronavirus M protein ectodomains, and determined the secondary structure of specific peptides using circular dichroism (CD) spectroscopy. Enzyme-linked immunosorbent assay (ELISA) was utilized to detect IgG responses against the synthesized peptides in clinical samples. To evaluate the immunogenicity of peptide vaccines, BALB/c mice were intraperitoneally immunized with peptide-keyhole limpet hemocyanin (KLH) conjugates adjuvanted with incomplete Freund’s adjuvant (IFA). The humoral and T-cell immune responses induced by peptide-KLH conjugates were assessed using ELISA and ELISpot assays, respectively. The efficacy of the S2M2-30-KLH vaccine against SARS-CoV-2 variants was evaluated in vivo using the K18-hACE2 transgenic mouse model. The inhibitory effect of mouse immune serum on SARS-CoV-2 virus replication in vitro was evaluated using microneutralization assays. The subcellular localization of the M protein was evaluated using an immunofluorescent staining method, and the Fc-mediated antibody-dependent cellular cytotoxicity (ADCC) activity of the S2M2-30-specific monoclonal antibody (mAb) was measured using an ADCC reporter assay.

**Results:**

Seroconversion rates for ectodomain-specific IgG were observed to be high in both SARS-CoV-2 convalescent patients and individuals immunized with inactivated vaccines. To assess the protective efficacy of the M protein ectodomain-based vaccine, we initially identified a highly immunogenic peptide derived from this ectodomain, named S2M2-30. The mouse serum specific to S2M2-30 showed inhibitory effects on the replication of SARS-CoV-2 variants *in vitro*. Immunizations of K18-hACE2-transgenic mice with the S2M2-30-keyhole limpet hemocyanin (KLH) vaccine significantly reduced the lung viral load caused by B.1.1.7/Alpha (UK) infection. Further mechanism investigations reveal that serum neutralizing activity, specific T-cell response and Fc-mediated antibody-dependent cellular cytotoxicity (ADCC) correlate with the specific immuno-protection conferred by S2M2-30.

**Discussion:**

The findings of this study suggest that the antibody responses against M protein ectodomain in the population most likely exert a beneficial effect on preventing various SARS-CoV-2 infections.

## 1 Introduction

The severe acute respiratory syndrome coronavirus 2 (SARS-CoV-2) viruses undergo constant mutation, leading to the emergence of new variants that can evade population immunity. Recent investigations have revealed a significant reduction in the efficacy of currently licensed SARS-CoV-2 vaccines against highly transmissible Omicron variants including the emerging XBB, BQ.1 and JN.1 strains (1–6). The persistent threat posed by SARS-CoV-2 to public health necessitates the explorative research of conserved antigens and the development of a vaccine capable of conferring cross-protection.

Membrane (M) protein is the most abundant viral structural protein of SARS-CoV-2 and plays versatile and pivotal roles in directing virion assembly, morphogenesis, and antagonizing mitochondrial antiviral signaling protein (MAVS)-mediated interferon responses (7–9). It has been shown that the M protein ectodomain of human coronavirus NL63 participates in viral attachment to host receptors, facilitating viral entry into host cells (10). The SARS-CoV-2 M protein consists of an N-terminal extracellular domain, three transmembrane helices interconnected by an extracellular loop and an intracellular loop, and a C-terminal intracellular domain (9, 11). Within the M protein, only ectodomain has the potential to induce a protective antibody response. The amino acid sequences of M proteins from sarbecovirus strains exhibit a high degree of homology (12, 13), implying that the immune response targeting this antigen could be cross-protective. It has been reported that a majority of SARS-CoV-2-infected patients produce N-terminal ectodomain-specific immunoglobulin (Ig) G antibodies during their convalescent phase (14, 15). Some B cell epitopes have also been identified in the M proteins of both SARS-CoV-1 and SARS-CoV-2 (16, 17). This study further assessed the seroconversion rate against N-terminal ectodomain in individuals who have been immunized with inactivated vaccines (18–20) and attempted to elucidate effector functions of its specific immunity by utilizing the human angiotensin-converting enzyme 2 (hACE2)-transgenic mouse model.

In this study, we have identified a highly immunogenic peptide S2M2-30 derived from M protein ectodomain. This peptide, when conjugated with keyhole limpet hemocyanin (KLH) carrier protein, induced a strong peptide-specific antibody response and cellular response, while the majority of other shorter peptides designed in this study did not show any immunogenicity. Immunization of human angiotensin-converting enzyme 2 (hACE2)-transgenic mice with S2M2-30-based immunogen demonstrated its effectiveness in providing protective immunity. The findings from this study would shed light on the authentic functions of M protein-specific population immunities.

## 2 Materials and methods

### 2.1 Human subjects

In this study, we recruited vaccinees from a prospective cohort at the Third Affiliated Hospital of Sun Yat-sen University in Guangzhou, China, all participants received the inactivated SARS-CoV-2 vaccine BBIBP-CorV (BBIBP-CorV, Sinopharm, Beijing) or CoronaVac (Sinovac Life Sciences, Beijing, China) on day 0, 28 and 270. Serum specimens from vaccinees who received two doses of the CoronaVac vaccine (n = 58) and vaccinees who received three doses of the CoronaVac vaccine (n = 30) were collected 14 days after the last immunization. Convalescent serum samples from coronavirus disease 2019 (COVID-19) patients (n = 15) were collected 14 days after release from hospitalization. Our study also included serum samples from child outpatients (n = 28) in mid-2019 for negative control use in serological tests. All serum samples were heat-inactivated at 56°C for 30 min before testing.

### 2.2 Cell lines and SARS-CoV-2 strains

VeroE6 cell line with transmembrane serine protease 2 (TMPRSS2) overexpression was purchased from the Japanese Collection of Research Bioresources (JCRB) Cell Bank and maintained in Dulbecco’s modified Eagle’s medium (DMEM) (Gibco, Cat. #11965092) supplemented with 10% heat-inactivated fetal bovine serum (FBS) (Gibco, Cat. #16140071), 100 U/ml penicillin, 100 μg/ml streptomycin (P/S) (Gibco, Cat. #15140122) and 1mg/ml G-418 (MCE, Cat. #HY-17561). A549 with TMPRSS2 and human ACE2 overexpression cell line that was used for immunostaining was purchased from InvivoGen company (Cat. #a549-hace2tpsa) and maintained in DMEM supplemented with 0.5 μg/ml of Puromycin (Thermofisher, Cat. #A1113803), 300 μg/ml of Hygromycin (Thermofisher, Cat. #10687010), 10% FBS and P/S. The following authentic SARS-CoV-2 viruses including HKU-001a (GenBank: MT230904), B.1.1.7/Alpha (UK) (GenBank: OM212469), B.1.617.2/Delta (GenBank: OM212471), Omicron/BA.1 (GenBank: OM212472), Omicron BA.5 (GISAID: EPI_ISL_13777658) and Omicron BQ.1.1 (GISAID: EPI_ISL_16342297) viruses were isolated from nasopharyngeal swabs of laboratory-confirmed COVID-19 patients and were used in this study. All the viruses were sub-cultured and tittered in plaque assay using the VeroE6-TMPRSS2 cell line. Each virus sample was aliquoted and stored at - 80°C for further use. All authentic SARS-CoV-2 related experiments were performed in the Biosafety Level 3 (BSL-3) Facility at the Department of Microbiology, The University of Hong Kong under the Standard Operation Procedures (SOPs).

### 2.3 Laboratory mouse models

Six-to-eight-week-old female BALB/c mice were obtained from the Hunan SJA Laboratory Animal Co., Ltd. and maintained with access to food and water ad libitum in a clean room kept at 22 ± 2°C and 50 ± 5% humidity, under a 12 h -12 h light-dark cycle. All animal experiments were performed as per the China Public Health Service Guide for the Care and Use of Laboratory Animals. Mice experiment protocols were approved by the Institutional Animal Care and Use Committee from the College of Biology, Hunan University (Approval Code: HNUBIO202202002).

Six-to-eight-week-old K18-hACE2 transgenic mice were purchased from The Jackson Laboratory (US) and kept in cages with individual ventilation under 65% humidity and an ambient temperature of 21 - 23 °C and a 12 h - 12 h day-night cycle for housing and husbandry in Centre for Comparative Medicine Research (CCMR) of The University of Hong Kong. All mice were housed and bred in our BSL-3 facility (21) and given access to standard pellet feed and water ad libitum.

### 2.4 M protein structure prediction with AlphaFold-2

The amino acid sequences of coronavirus M proteins were obtained from UniProt: P59596, VME1_SARS; P0DTC5, VME1_SARS2 and K9N7A1, VME1_MERS1, and the predicted structural models were generated using the online protein structure prediction tool AlphaFold (version 2) (https://colab.research.google.com/github/sokrypton/ColabFold/blob/main/AlphaFold2.ipynb). The models with the highest ranking were chosen for visualization using ChimeraX software (UCSF, https://www.cgl.ucsf.edu/chimerax/).

### 2.5 Peptides synthesis

The set of peptides listed in **Table 1** was synthesized by a standard solid-phase Fmoc (9-fluorenyl methoxycarbonyl) method in Shanghai Top-Peptide Biochem Co., Ltd. Peptides were purified to homogeneity (purity of > 95%) by high-performance liquid chromatography and identified by laser desorption mass spectrometry. A monosaccharide N-acetylglucosamine was linked to the fifth asparagine of the Gly-S2M2-20-Mid peptide.

**Table 1:** Amino acid sequences of the synthetic peptides from coronavirus M protein ectodomains.

Viruses	Peptide names	Amino acid sequences
SARS-CoV-2	S2M2-20	ADSNGTITVEELKKLLEQW
	S2M8-24	ITVEELKKLLEQWNLVI
	S2M11-30	EELKKLLEQWNLVIGFLFLT
	S2M2-30	ADSNGTITVEELKKLLEQWNLVIGFLFLT
	S2M74-77	NWIT
	S2M2-20-Mid	ADSNGTITVEELKKLLEQWGSGSNWIT
	Gly-S2M2-20-Mid	ADSN(gly)GTITVEELKKLLEQWGSGSNWIT
	S2M11-20	EELKKLLEQW
	S2M21-30	NLVIGFLFLT
SARS-CoV-1	S1M2-19	ADNGTITVEELKQLLEQW
MERS-CoV	MSM2-19	SNMTQLTEAQIIAIIKDW

The (gly) in the amino acid sequence of Gly-S2M2-20-Mid indicates the addition of a monosaccharide at N5.

### 2.6 Circular dichroism spectroscopy

The secondary structures of the synthetic peptides in the solid state were confirmed by far UV CD spectroscopy using a JASCO J-815 spectrometer (JASCO Analytical Instruments, Easton, MD). The lyophilized peptide powders were spread on 2-cm diameter cylindrical quartz glass, and the spectra of samples were recorded over a wavelength range of 190 - 260 nm at a scanning speed of 10 nm/min and a time constant of 0.5 s. An average of three scans was reported. The data of CD spectra was analyzed using CDNN CD spectral deconvolution software package (version 2.1, Applied Photophysics Ltd., Leatherhead, UK), and the proportion of each secondary structure (α-helix, β-antiparallel, β-turn, unordered random coli) was calculated by running “deconvolute”. The final spectra were expressed as molar circular dichroism Δϵ (L·mol^−1^·cm^−1^) per residue or molar absorption coefficient ϵ (L·mol^−1^·cm^−1^) per residue.

### 2.7 Preparation of peptide-KLH conjugates

The conjugations of peptides to KLH protein carrier were performed according to the manufacturer’s instruction of ReadiLink KLH Conjugation Kit (AAT Bioquest, Cat. #5502). In brief, 1 mg soluble peptide was dissolved in 0.5 ml phosphate-buffered saline (PBS) and mixed with 1 mg KLH in 0.5 ml PBS. The peptides S2M11-30, S2M2-30, and MSM2-19, which exhibited poor solubility in PBS, were dissolved in 0.5 ml of PBS containing 5% dimethyl sulfoxide (DMSO, Solarbio, Cat. #D8371) and subsequently mixed with KLH. Fifty μl 1% (w:v) of 1-ethyl 1-3-[dimethylaminopropyl] carbodiimide hydrochloride (EDC) was immediately added to the mixture of peptide and KLH, then gently mixed and incubated at room temperature for 2 h. Spin desalting columns (7K MWCO) were used to purify the conjugates and to remove the non-reacted crosslinker. The collected conjugate samples were sterilely filtered by using a 0.22 μm filter membrane and then stored at -80°C.

### 2.8 Mice immunization and infection experiments

Six-eight-week-old female BALB/c mouse models were used for immunization. The immunization regimen is depicted in **Supplementary Figure S1A**. Mice (n = 6 per group) were intraperitoneally immunized three times with 10 μg per dose of peptide-KLH emulsified with Incomplete Freund’s Adjuvant (IFA, Sigma, Cat. #F5506), or 10 μg per dose of unconjugated KLH emulsified with IFA, or two times with 10 μg per dose of receptor binding domain (RBD)-hFc (Sino Biological Inc., Cat. #40592-V02H) emulsified with IFA. Blood samples were collected one day before prime immunization and 2 weeks after each immunization. All groups of mice were sacrificed by cervical dislocation on day 63, and then blood samples and spleens were collected for subsequent experiments.

Six-eight-week-old male K18-hACE2 transgenic mice were used for the SARS-CoV-2 challenge experiment (21) as depicted in **Supplementary Figures S1B, C**. The sequential immunization regimen is described in **Supplementary Table S1**. Ten μg per dose of peptide-KLH emulsified with IFA, 10 μg per dose of RBD-hFc emulsified with IFA, or 10 μg per dose of unconjugated KLH emulsified with IFA were employed in this immunization experiment. Blood samples were taken one day before prime immunization and 10 days post the second, fourth, and fifth immunizations. Two weeks after the last immunization, mice were anesthetized with Ketamine (200 mg/kg) (purchased from CCMR, HKU) and Xylazine (10 mg/kg) (purchased from CCMR, HKU), followed by intranasal inoculation with 10^4^ plaque forming unit (PFU) of B.1.1.7/Alpha (UK) or 10^5^ PFU of B.1.1.529/Omicron BA.1. Lung samples were collected for viral RNA copy detection on 2 days after B.1.1.7/Alpha (UK) infection. Body weight and survival rate were recorded daily for 14 days after B.1.1.529/Omicron BA.1 infection. Nasal irrigation samples were collected for viral RNA copy detection on day 4 after B.1.1.529/Omicron BA.1 infection. All SARS-CoV-2 infection experiments were performed in the BSL-3 facility and were approved by the Committee on the Use of Live Animals in Teaching and Research (CULATR) of The University of Hong Kong.

The infected cell culture or animal samples were lysed by using RLT buffer (Qiagen, Cat. #79216), followed by viral RNA extraction using RNeasy Kit (Qiagen, Cat. #74004) according to the manufacturer’s protocol. The viral copies were determined by real-time reverse transcription quantitative polymerase chain reaction (RT-qPCR) assay in accordance with the protocol by Takara (Cat. #RR086A). Among the validated real-time reverse transcription-PCR (RT-PCR) assays, measurement of RNA-dependent RNA polymerase (RdRp) copies has shown superior analytical sensitivity and specificity compared to other structural proteins, such as the envelope and nucleocapsid proteins (22–26). The copy numbers of reverse-transcripts from cell cultures and mice organ samples were normalized with the expression level housekeeping gene of beta-actin by using the 2(-Delta Delta C(T)) method (27). The primers used in this study targeting SARS-CoV-2 RdRp gene include Forward primer: 5’-CGCATACAGTCTTCAGGCT-3’ and Reverse primer: 5’-GTGTGATGTTGAWATGACATGGTC-3’, and the primers targeting beta-actin gene include Forward primer: 5’-ACGGCCAGGTCATCACTATTG-3’ and Reverse primer: 5’-CAAGAAGGAAGGCTGGAAAAG-3’.

### 2.9 Serological analysis

Titers of peptide-specific and RBD-specific serum IgG were determined by using enzyme-linked immunosorbent assay (ELISA). The 96-well immuno-plates (JET BIOFIL, Cat. #FEP100096) were coated with 100 ng per well of peptides (**Table 1**) or purified Wuhan-Hu-1 RBD protein (Wuhan-Hu-1 RBD (Arg319 - Phe541), GenBank: QHD43416.1), at 4°C, overnight. The immuno-plates were blocked with 3% bovine serum albumin (BSA, Sigma, Cat.#0336-50ML) in 1 × PBS supplemented with 0.1% (v:v) Tween-20 (PBST) at 37°C for 1 h, followed by incubation with three-fold serially diluted serum samples starting from 1:100 to 1:218700 at 37°C for 1 h. Immuno-plates were then washed three times with 0.1% PBST and incubated with 0.2 μg/ml goat anti-mouse IgG conjugated with horseradish peroxidase (HRP) (ABclonal Technology, Cat.#AS003) in blocking buffer at 37°C for 1 h. Immuno-plates were washed four times and TMB substrate (Solarbio, Cat.#K8160) was added for color development reaction. The reaction was stopped by adding 1 M sulfuric acid and the absorbance was measured at 450 nm. The cut-off for seropositivity was set as mean + 2 × standard derivation (SD) value of the Preimmune serum control group and indicated by a dashed line. The HRP-conjugated goat anti-mouse IgG1 (ABclonal Technology, Cat. #AS066), the HRP-conjugated goat anti-mouse IgG2a (ABclonal Technology, Cat. #AS065), or the HRP-conjugated goat anti-mouse IgM (Sangon Biotech, Cat. #D110103-0100) was used to detect the bound serum IgG1, IgG2a, IgM antibodies.

The ELISA experiment procedure for titrating specific antibodies of human serum samples was slightly changed. In brief, High-binding 96-well immuno-plates (Thermo, Cat. #442404) were coated with 50 μl per well, 2 μg/ml of peptide antigens in 1 × carbonate-bicarbonate buffer at 37°C overnight until the coating was dry. On the following day, the wells were blocked with 300 µl blocking buffer, containing 5% skim milk powder (BBI, Cat. #A600669-0250) in PBST, at room temperature for 1 h. Human serum samples were diluted 300 and 900 times for binding to antigen and incubated at room temperature for 2 h. After washing 3 times with washing buffer, the secondary anti-human IgG antibody conjugated to HRP (Jackson ImmmunoResearch, Cat. #109-035-088) dilution was used for incubation at room temperature for 1 h. Immuno-plates were washed four times and TMB substrate (Solarbio, Cat. #K8160) was added for color development reaction. The reaction was stopped by adding 1 M sulfuric acid, absorbance was measured at 450 nm using a microplate spectrophotometer (Agilent, BioTek, U.S.), and the OD values were used to calculate the area under curve (AUC) using GraphPad Prism 8.0.

### 2.10 Microneutralization assays

Serially diluted immune serum samples were pre-incubated with 200 PFU/ml SARS-CoV-2 at 37°C for 1 h. The combined serum samples were composed of serially diluted (1:200, 400, or 800) RBD-induced immune serum and serially diluted (1:8 or 16) peptide-KLH-induced or KLH-induced immune serum samples. The serum-virus mixtures were then inoculated in the confluent VeroE6-TMPRSS2 cell culture for 1 h. The cell cultures were washed twice using sterile PBS and maintained in serum-free culture medium at 37°C for 48 h. The culture supernatants were harvested for reverse transcription qPCR detection of viral load.

We also conducted immuno-fluorescent staining of wild-type HKU-001a-infected Vero-E6-TMPRSS2 cells for the evaluation of serum neutralizing activity. Cells were seeded in 96-well cell culture plates (Corning, Cat. #3904) at a density of 20,000 cells/well and incubated at 37°C, 5% CO_2_ overnight. The serum samples were 2-fold serially diluted with an initial dilution factor of 1:2, then each serum diluent sample was mixed with 200 PFU of wild-type HKU-001a and incubated at 37°C for 1 h. Virus-serum mixtures were added to cell culture plates for infection, the virus solution alone was used as a positive control for infection. After 24 h incubation, the supernatant was removed and the cells were fixed with 4% paraformaldehyde at room temperature for 15 min. The cell culture was washed three times with sterile PBS and permeabilized with 100 μl 0.5% TritonX-100 (Sigma, Cat. #93443) at room temperature for 15 min. After washing three times with PBS, the cells were incubated with 1:4000 fold diluted rabbit anti-SARS-CoV-2 N protein-specific IgG (Abcam, Cat. #ab271180) at room temperature for 1 h. After washing three times with PBS, 1:100 fold diluted Goat anti-Rabbit IgG (H+L) Cross-Adsorbed Secondary Antibody (Alexa Fluor 488, ThermoFisher, Cat. #R37116) was then added and incubated at room temperature for 1 h. The fluorescently stained cell culture plate was imaged using Sapphire Biomolecular Imager (Azure Biosystems, U.S.). Quantitative analysis of the fluorescence intensity from each well was performed by using the ReadPlate 3.0 module in ImageJ software. The SARS-CoV-2 inhibition ratio was calculated using the following formula, SAR-CoV-2 Inhibition (%) = (1- fluorescence intensity of each well/fluorescence intensity of virus only well) × 100%.

### 2.11 Immunofluorescent staining analysis

Human embryonic kidney cell line (HEK293T) was transfected with a recombinant plasmid for the expression of the M-green fluorescent protein (M-GFP) fusion protein, which consists of the full-length M protein from wild-type HKU-001a and GFP. The HEK293T cells were seeded at a density of 5 × 10^5^ cells per well and were grown at 37°C overnight on glass coverslips in a 24-well cell culture plate (JET BIOFIL, Cat. # TCP011006). Then, cells were transfected with 2 μg eucaryotic recombinant plasmid pCMV3-C-GFPSpark (C-GFPSpark tag, Sino Biological Inc., Cat. #VG40608-ACG) encoding M-GFP. Six μl Lipofectamine 3000 (Thermo Fisher, Cat. #L3000001) was used for encapsulating 2 μg plasmid. The lipid complex was wise-dripped into each well and incubated at 37°C, 5% CO_2_ for 6 h. Then the cells were cultured in DMEM medium supplemented with 10% FBS at 37°C, 5% CO_2_ for an additional 24 h. The successful expression of the M-GFP fusion protein was confirmed by observing green fluorescence using an inverted phase contrast fluorescence microscopy (Nikon, Cat. #TS2R-FL, Japan). The 1,1’-dioctadecyl-3,3,3’,3’-tetramethylindocarbocyanine perchlorate (DIL, Solarbio, Cat. #D8700) and 2-(4-Amidinophenyl)-6-indolecarbamidine dihydrochloride (DAPI, Solarbio, Cat. # C0060) were used for staining cell membrane and cell nucleus, respectively.

For immunostaining of the transfected cells, the HEK293T cells over-expressing M-GFP fusion protein were fixed using 4% paraformaldehyde (Solarbio, Cat. #P1110) in tris-buffered saline (TBS) at 4°C for 15 min, permeabilized using 0.2% Triton X-100 (Sigma, Cat. #93443) in TBS at room temperature for 20 min, blocked using 10% normal goat serum (Solarbio, Cat. #SL038) at room temperature for 1 h, and incubated with 1:100 diluted S2M2-30-KLH antiserum at 4°C overnight. After washing three times with PBS, 5 min each time, the cells were incubated with Cy3 Goat Anti-Mouse IgG H&L (ABclonal, Cat. #AS008) at room temperature for 1 h. After washing three times with PBS, the cells were stained with 2 μg/ml DAPI at room temperature for 5 min. The stained cells on the coverslips were imaged using a laser scanning confocal microscope (ECLIPSE, Nikon, Japan).

For immunostaining of the infected cell cultures, pre-seeded A549-TMPRSS2-ACE cells were infected with SARS-CoV-2 wild-type HKU-001a or Omicron/BA.5 variant with a MOI of 2. Two h post-infection, the cell culture supernatant was discarded, and cells were fixed and blocked. The cells were incubated with S2M2-30-KLH-specific immune serum at room temperature for 1 h, then were washed 3 times using PBST, followed by an incubation with the secondary antibody Goat Anti-Mouse IgG H&L (Alexa Fluor 488, Abcam, Cat. #ab150113). The phalloidin-iFluor dye (Abcam, Cat. #ab176759) and DAPI were used for staining F-actin and nucleus in cells, respectively. The images were taken by Carl Zeiss LSM880 system Confocal Laser Scanning Microscope (Dublin, CA, USA).

### 2.12 Enzyme-linked immunosorbent spot assay

Mice were sacrificed 3 weeks after the last immunization and soaked in 75% ethanol for a while before the isolation of spleen samples. The spleen organs were aseptically isolated and were cut into small pieces in cell culture dishes using sterilized ophthalmic scissors and homogenated using the piston end of the syringe, then the samples were transferred into 40 μm cell strainer and washed with 5 - 10 ml Roswell Park Memorial Institute (RPMI) 1640 medium (Gibco, Cat. #11875119). The suspensions of single splenocytes were immediately transferred to a 15 ml sterile centrifugation tube and centrifuged at 300 × g at 4°C for 10 min. After centrifugation, lymphocytes were isolated by using a Mouse Spleen Lymphocyte Separation Kit (Solarbio, Cat. #P8860). The harvested lymphocytes were resuspended in RPMI 1640 medium at a cell density of around 10^8^ cells per ml and then were seeded in ELISpot plates. After restimulation using sterile peptide solution containing S2M2-20 or S2M2-30, interferon (IFN)-γ-secreting cells were detected using a mouse IFN-γ ELISpot Kit (BD, Cat. #551083, U.S.), and interleukin (IL)-4-secreting cells were detected using a mouse IL-4 ELISpot Kit (DAKEWE, Cat. #2210403). Concanavalin A (ConA, Sigma, Cat. #C2272) at a final concentration of 2 μg/ml was used as a positive control for reactivating T lymphocytes. Spots were counted using an ELISpot automatic plate reader (AID Elispot Reader, AID, Germany).

### 2.13 Antibody-dependent cellular cytotoxicity reporter assay

The Jurkat–Lucia NFAT-CD16 cell line stably expressing human FcγRIIIa of Valine 158 allotype (Invivogen, Cat. #jktl-nfat-cd16) and transfected HEK293T cells expressing M-GFP were used as effector cells and target cells, respectively, in ADCC reporter assays. The expression of recombinant M-GFP fusion protein was confirmed by observing green fluorescence in cells using an inverted phase contrast fluorescence microscopy. An S2M2-30-specific chimeric monoclonal antibody (mAb), 3M1C11, comprising a human IgG1 Fc domain and CR6261 IgG1 mAb was diluted using the complete DMEM medium, and the dilutions were added to the target cell cultures in a 96-well cell culture plate at 37°C for 2 h. The 1.5 × 10^5^ effector cells resuspended in RPMI 1640 medium were also added for incubation at 37°C, 5% CO_2_, for 20 h.

ADCC activity was determined by measuring the activity of luciferase secreted from the activated Jurkat–Lucia NFAT-CD16 cells. Thirty µl supernatant was collected from each well and mixed with 25 µl substrate QUANTI-Luc 4 Lucia detection reagent (Invivogen, Cat. #rep-qlc4lg1) in a black 96-well flat-bottom assay plate and the plate was read using a multimode microplate reader (PerKinElmer, EnSight). The fold of change (ADCC induction) was calculated with the relative light unit (RLU) using the following formula, (RLU of test well - RLU of well with only complete medium)/(RLU of well where the supernatant collected from the cell cultures without the addition of mouse serum or mAb − RLU of well with only complete medium).

### 2.14 Preparation, expression, and purification of S2M2-30-specific mAb

S2M2-30-specific mAbs were isolated by using single-cell sorting of S2M2-30-reacted memory B cells that were collected from S2M2-30-KLH-immunized mice. In brief, six-to-eight-week-old female BALB/c mice were immunized intraperitoneally four times with 30 μg S2M2-30-KLH per dose emulsified with IFA, at a two-week interval. Two weeks after the last immunization, serum samples were collected for antibody titration using ELISA. S2M2-30-specific antibody titers above 6 × 10^4^ were deemed eligible for the next single-cell sorting. Then, the immunized mice were sacrificed and the single splenocytes were prepared. S2M2-30-specific memory B cells were isolated using flow cytometric cell sorting. Gene fragments encoding the Ig variable region of the heavy (VH) and Ig variable region of the light (VL) chains were PCR-amplified, purified, and sequenced with 10 mM forward and reverse specific primers. Then, these genes were cloned into the recombinant pDNA3.4 expression vector, in which the VH gene was genetically fused to the 5’ end of the gene fragment encoding human IgG1 constant heavy (CH) 1 to CH3 domains, while the VL gene was linked to the 5’ end of the gene fragment encoding human CL of κ class in open reading frames. The chimeric heavy and light chains were expressed by co-transfected HEK293F suspension cells. The IgG molecules were purified from cell culture supernatant by using Protein A resin. The purified IgG samples were characterized by using SDS-PAGE analysis and ELISA.

### 2.15 Statistical analysis

Statistical analysis was performed using GraphPad Prism 9 software v9.1.1. Unpaired two-tailed Student’s *t*-tests were employed for the data analysis pertaining to titration of specific antibodies, measurement of cellular response, and determination of copy numbers of viral RdRp gene in mice lung homogenate samples and nasal irrigation samples. The peptide-specific serum titers as continuous outcome variables that were affected by two independent predictor variables, including the individual variance and the number of vaccinations, were compared among the indicated sample groups by using the two-way ANOVA method with Tukey’s multiple-comparison test. Comparisons of SARS-CoV2 RdRp RNA copies from different groups in microneutralization assays, comparisons of peptide binding activity, and comparisons of the ADCC activities of test groups were performed using multiple *t*-tests. Differences were considered to be statistically significant when *P *< 0.05. The ‘ns’ indicate no significant difference between groups in comparison.

## 3 Results

### 3.1 Design of peptides derived from M protein ectodomain

**Figure 1:**
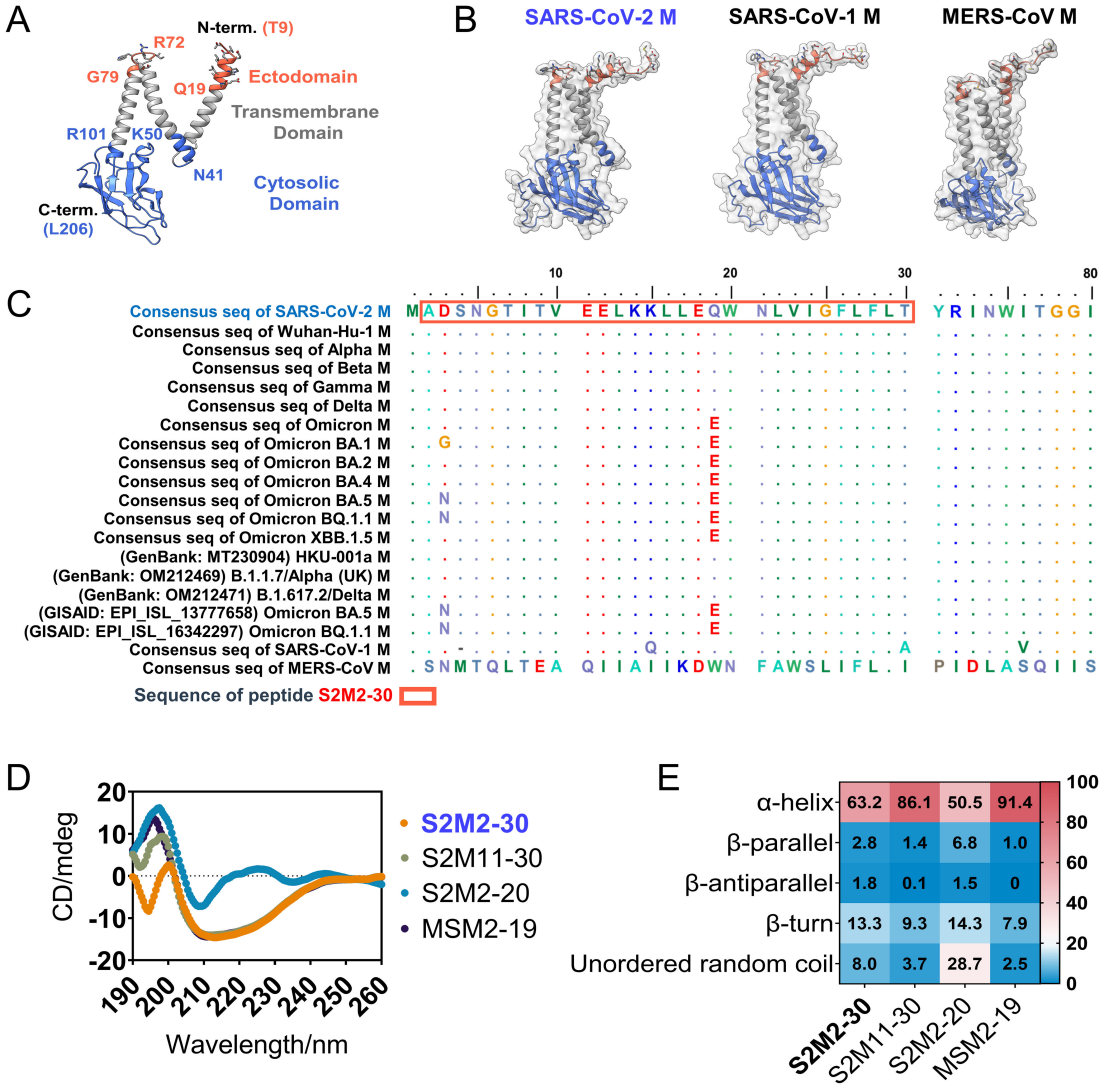
Structure and sequence analysis of M protein ectodomain. **(A)** The SARS-CoV-2 M protein protomer is shown with a ribbon model (PDB ID: 7VGR). **(B)** The AlphaFold-2-predicted structures of the full-length M proteins from SARS-CoV-2, SARS-CoV-1, and MERS-CoV. **(C)** Amino acid sequence alignment of M protein ectodomains. **(D)** The circular dichroism spectra detection of S2M2-20, S2M11-30, S2M2-30, and MSM2-19 for secondary structure analysis. **(E)** The output data of circular dichroism spectra analysis indicates the proportion of each type of protein secondary structure.

As the structure of the extreme N-terminal fragment remains unsolved (9) (**Figure 1A**), we employed Alphafold-2 to predict the conformation of the folded M polypeptides derived from SARS-CoV-2, SARS-CoV-1, and middle east respiratory syndrome coronavirus (MERS-CoV). These structural models suggest that ectodomain peptides are likely to adopt a flexible conformation (**Figure 1B**). The alignment of amino acid sequences revealed a high level of conservation in the ectodomains of SARS-CoV-2 M proteins, with mutations D3G/N and Q19E exclusively present in the Omicron variants (**Figure 1C**). We synthesized multiple peptides spanning residues 2 - 30 of the M protein from SARS-CoV-2, and peptides consisting of residues 2 -19 of the M protein from SARS-CoV-1 and MERS-CoV (**Table 1**). These peptides were used in the subsequent serological tests and mouse immunization experiments. The glycosylation at N5 of M protein is believed to impact antibody responses targeting M protein ectodomain. Therefore, we also included a peptide, named Gly-S2M2-20-Mid, with a monosaccharide N-acetylglucosamine linked at N5. A circular dichroism detection showed that secondary structures of peptides S2M2-30, S2M11-30, S2M2-20, and MSM2-19 predominantly present as alpha-helices (**Figures 1D, E**).

### 3.2 M protein ectodomain-specific IgG response was detected in individuals vaccinated with CoronaVac inactivated vaccines

**Figure 2:**
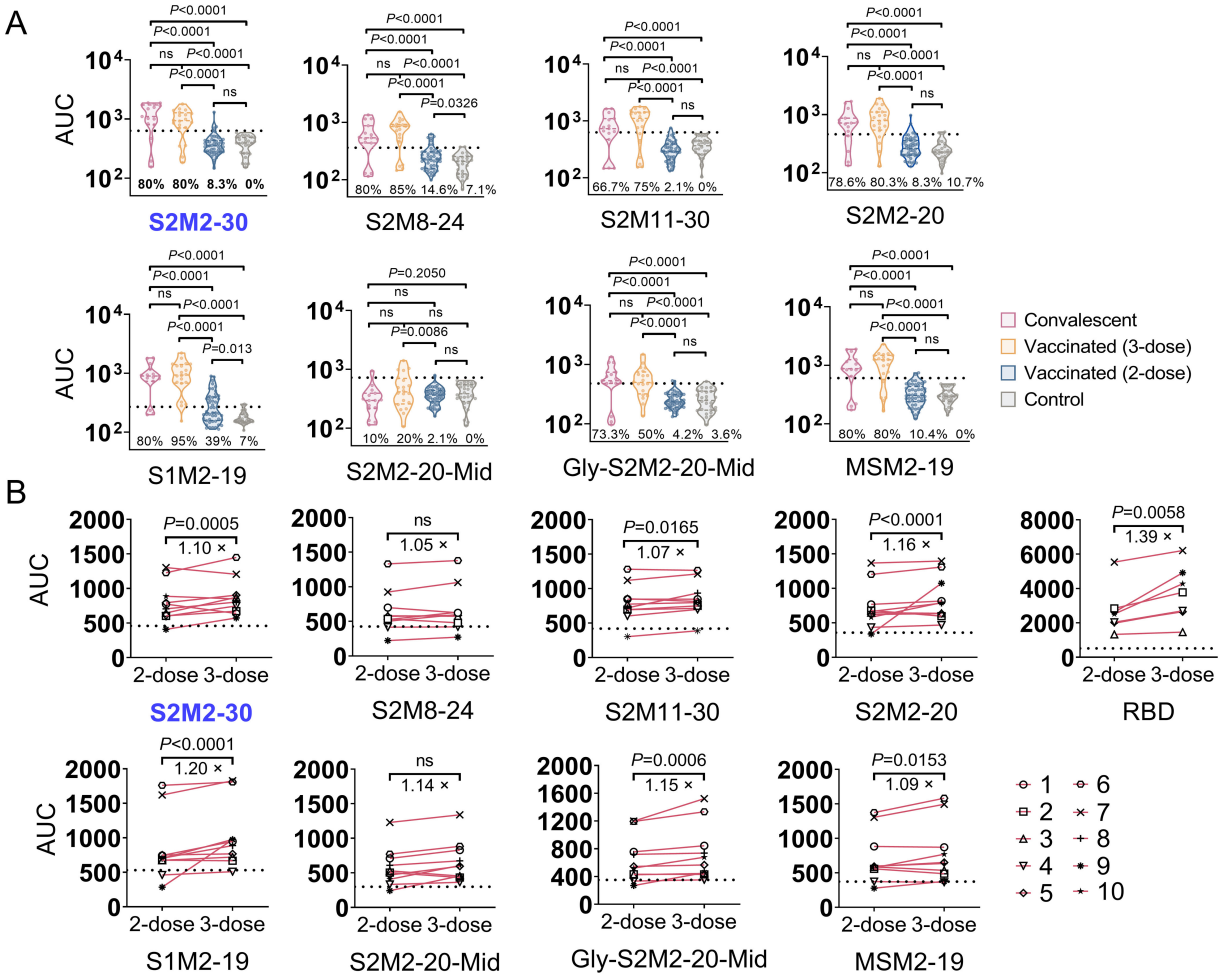
Seroconversion rates for M peptide-specific IgG responses in the study population. **(A)** The area under curve (AUC) of specific serum IgG. Each point represents the individual’s specific antibody titer. Serum samples were collected from convalescent COVID-19 patients (n = 15), individuals immunized with 2-dose (n = 48) or 3-dose (n = 20) of CoronaVac inactivated vaccine, and outpatient children in mid-2019 (n = 26). Dashed lines indicate the mean + 2 × SD of the group of serum collected in mid-2019. AUC values above dashed lines are regarded as positive results. **(B)** Longitudinal serological analysis of the IgG response in individuals receiving 2-dose and 3-dose vaccinations. Dashed lines represent background levels, indicating mean + 2 × SD values from the negative control (n = 2). Statistical significance between groups was assessed using an unpaired *t*-test for panel **(A)** while two-way ANOVA for panel **(B)**. *P* values less than 0.05 are considered statistically significant. The ‘ns’ indicates not significant.

To assess the incidence of seroconversion for M protein ectodomain-specific IgG in the population, we collected 10 serum samples from each study group, including unvaccinated convalescent COVID-19 patients who were confirmed in 2020 using SARS-CoV-2 reverse transcription polymerase chain reaction (PCR), individuals who received two doses of CoronaVac inactivated vaccines and remained uninfected, and outpatient children at Beijing Children’s Hospital in mid-2019 who were previously SARS-CoV-2 infection naïve. The convalescent serum exhibited significantly stronger binding activities towards all peptides in the tests, in comparison to either the vaccine-induced or pre-pandemic serum specimens (**Supplementary Figure S2**). In another independent serological assay, the sample size of each group was increased to improve the precision of estimates. Furthermore, an additional 3-dose group consisting of 20 serum samples collected from individuals who received three consecutive immunizations with the CoronaVac inactivated vaccine was included. We observed a higher seroconversion rate in the 3-dose group compared to both the convalescent group and the 2-dose group (**Figure 2A**). A longitudinal serological analysis of vaccine recipients also revealed an increase in peptide-specific serum IgG titer after the administration of a third dose of CoronaVac (**Figure 2B**).

### 3.3 S2M2-30-KLH conjugate elicited significant humoral and cellular immune responses in mice

**Figure 3:**
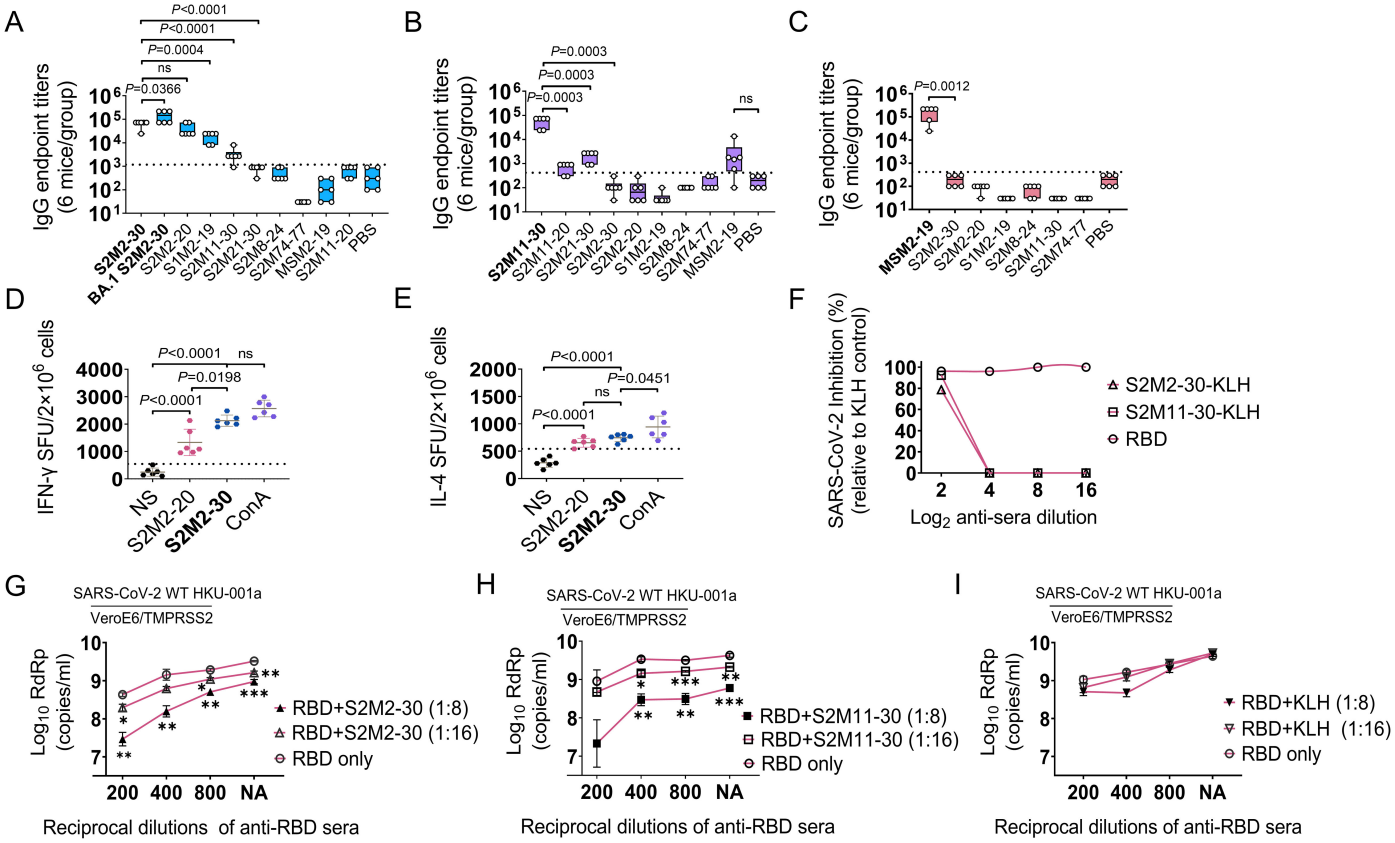
Peptide-specific immune response in BALB/c mice (n = 6). **(A-C)** IgG cross-reactivities with peptides in immune serum samples induced by S2M2-30-KLH, S2M11-30-KLH, or MSM2-19-KLH. The values on the Y-axis correspond to the reciprocal of the highest serum dilution showing a positive result. **(D, E)** Detection of IFN-γ-secreting and IL-4-secreting splenocytes. The “NS” below the X-axis indicates that splenocytes were not stimulated. ConA was used as a positive control. The abbreviation “SFU” on the left side of the Y-axis denotes spot forming units. **(F-I)** Microneutralization assays (n = 2). The “NA” below the X-axis in panels **(G-I)** means no addition of RBD-specific serum. All data are presented as mean ± standard error of the mean (SEM). The dashed lines in panels **(A-C)** represent the mean + 2 × SD of the PBS group. The dashed lines in panels **(D, E)** represent the mean + 2 × SD of the NS group. Statistical significance between compared groups was determined using an unpaired *t*-test for panels **(A-E)** while multiple *t*-test was used for panels **(G-I)**. *P* values less than 0.05 are considered statistically significant. The *, **, and *** represent *P* values less than 0.05, 0.01, and 0.001, respectively, and the ‘ns’ means not significant.

To evaluate and compare the immunogenicity of peptide vaccines, BALB/c mice were intraperitoneally immunized three times with peptide-KLH conjugates adjuvanted with incomplete Freund’s adjuvant (IFA) (**Supplementary Figure S1A**). KLH antigen and Wuhan-Hu-1 receptor-binding domain (RBD) antigen were used as the negative and positive controls, respectively, in the immunization experiments. Among all synthesized peptides derived from the SARS-CoV-2 M protein, only S2M2-30 and S2M11-30 induced IgG responses, with S2M2-30 exhibiting superior immunogenicity (**Supplementary Figure S3A**). Furthermore, the immune serum induced by S2M2-30 exhibited broader reactivities towards the tested peptides compared to other serum samples (**Figures 3A–C**). Comparable KLH-specific IgG titers were detected in all peptide-KLH immunization groups, indicating that immunization experiments were performed well (**Supplementary Figure S3B**). We also observed that IgG1 dominated the peptide-specific IgG responses (**Supplementary Figures S3C, D**). Despite being vaccinated with potent peptide vaccines, mice still mounted relatively weak IgM responses (**Supplementary Figure S3E**). We collected splenocytes from mice immunized with S2M2-30-KLH and S2M2-20-KLH, respectively, to evaluate T cell responses in the ELISpot assay. Compared to the unstimulated groups, significantly increased populations of IFN-γ-secreting lymphocytes and IL-4-secreting lymphocytes were observed upon re-stimulation with S2M2-20 peptide or S2M2-30 peptide (**Figures 3D, E**). The population of IFN-γ-secreting lymphocytes was larger following restimulation with S2M2-30 compared to S2M2-20 (**Figure 3D**). The microneutralization results showed that both S2M2-30-specific serum and S2M11-30-specific serum exerted inhibitory effects on the replication of authentic wild-type SARS-CoV-2 strain HKU-001a *in vitro* (**Figure 3F**). It is noteworthy that combinations of S2M2-30-specific serum and RBD-specific serum exhibited significant synergy that hinders the viral replication of wild-type HKU-001a replication *in vitro* (**Figures 3G–I**).

### 3.4 S2M2-30-specific immunity cross-inhibited SARS-CoV-2 variants replication *in vitro* and *in vivo*

**Figure 4:**
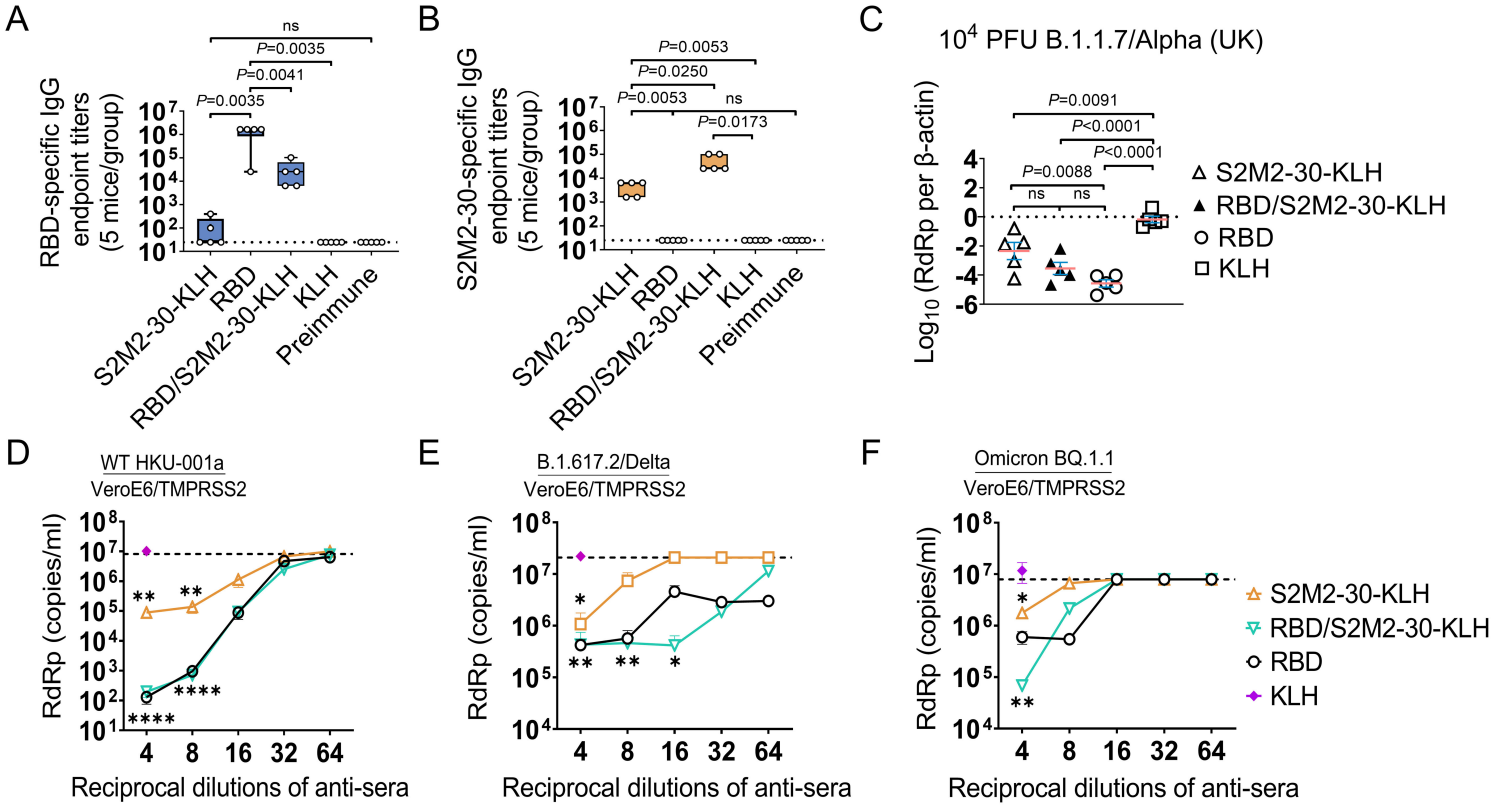
Immune response and protection in the immunized K18-hACE2-transgenic mice (n = 5). **(A, B)** The endpoint titers of serum IgG specific to Wuhan-Hu-1 RBD protein and S2M2-30 peptide. The values on the Y-axis represent the reciprocal of the highest serum dilution showing a positive result. The dashed lines represent the mean + 2 × SD of the Preimmune group. It is noteworthy that no significant difference between the S2M2-30-KLH and Preimmune groups in panel **(A)**. **(C)** Copy numbers of viral RdRp gene in mice lung homogenate samples were compared on the 2^nd day post intranasal infection with B.1.1.7/Alpha (UK). Copy numbers of all viral RdRp genes were normalized to β-actin housekeeping gene expression. **(D–F)** Microneutralization assays. **(D)** Wild-type HKU-001a, **(E)** B.1.617.2/Delta, and **(F)** Omicron BQ.1.1 strains were utilized to assess serum neutralizing activity. The dashed lines in panels **(D–F)** represent the mean - 2 × SD of the negative control group in which Preimmune serum was used for testing. All data are presented as mean ± SEM. *P* values less than 0.05 are considered statistically significant, and the ‘ns’ indicates no significance. The *, **, and **** represent *P* values less than 0.05, 0.01, and 0.001, respectively.

To assess the efficacy of the S2M2-30-KLH vaccine either alone or in combination with RBD vaccine, we devised an immunization schedule using the K18-hACE2 transgenic mouse model, in which a group of mice received two doses of RBD vaccine followed by three doses of S2M2-30-KLH vaccine, and two groups of mice were vaccinated with two doses of RBD vaccine and three doses of S2M2-30-KLH vaccine, respectively (**Supplementary Figure S1B**, **Supplementary Table S1**). Antibody titration results showed that both S2M2-30-KLH and RBD are robustly immunogenic in mice (**Figures 4A, B**). The sequential immunizations with RBD and S2M2-30-KLH resulted in an approximately 10-fold decrease in RBD-specific IgG titers compared to RBD immunization while leading to a roughly 10-fold increase in S2M2-30-specific IgG titers compared to the S2M2-30-KLH group (**Figures 4A, B**). To assess the cross-protection efficacy of the peptide vaccine, immunized mice were infected with a heterologous B.1.1.7/Alpha (UK) strain. Two days post-infection with 10^4^ PFU of B.1.1.7/Alpha (UK), mice from the S2M2-30-KLH group exhibited significantly lower lung viral loads compared to the KLH group, in one of which the decline in lung viral load was larger and comparable to the mean value of the RBD group, indicating strong *in vivo* cross-protection efficacy of S2M2-30-KLH (**Figure 4C**). The RBD, as a major neutralizing epitope, conferred robust sterile protection against B.1.1.7/Alpha (UK) infection, as evidenced by the substantial decrease in lung virus loads in the RBD group (**Figure 4C**). However, probably due to the markedly decreased RBD-specific antibody titers (**Figure 4A**), the RBD/S2M2-30-KLH group did not show any significant differences in viral loads compared to both the S2M2-30-KLH group and the RBD group (**Figure 4C**).

We also employed a heterologous B.1.1.529/Omicron BA.1 strain to test the vaccine efficacy. Due to the reduced pathogenicity of this Omicron strain in K18-hACE2 transgenic mice, a much higher infectious dose of 10^5^ PFU in inocula was used for intranasal infection in the challenge experiment (**Supplementary Figure S1C**). Four days post infection, only in the RBD/S2M2-30-KLH and RBD groups were viral loads in nasal irrigation samples markedly decreased (*P*<0.05) (**Supplementary Figure S4A**). Following infection, all groups of mice experienced considerable weight loss. The survival rates for the S2M2-30-KLH, RBD/S2M2-30-KLH, RBD, and KLH groups were 60%,100%, 75%, and 60%, respectively (**Supplementary Figures S4B, C**). Notably, while RBD/S2M2-30-KLH group demonstrated lower RBD-specific IgG titers compared to the RBD group (**Figure 4A**), it displayed a higher survival rate (**Supplementary Figure S4C**), suggesting that the additional S2M2-30-specific immunity may contribute to *in vivo* protection.

The results of microneutralization assays showed that S2M2-30-specific sera alone not only inhibited the replication of homologous HKU-001a but also cross-reacted with and inhibited the heterologous variants B.1.617/Delta and Omicron BQ.1.1 (**Figures 4D–F**). However, the antiviral synergistic effect as described in **Figure 3G** was not detected in these assays. The diminished antiviral effect observed in the RBD/S2M2-30-KLH group could be attributed to the reduced levels of RBD-specific IgG titers within this group (**Figure 4A**). It is noteworthy that, despite a 10-fold reduction in RBD-specific IgG titer, the RBD/S2M2-30-KLH group still exhibited a comparable neutralization activity to the RBD group (**Figures 4A, D–F**), suggesting that S2M2-30-specific immunity in the context of combined inoculations similarly contributes to *in vivo* antiviral function.

### 3.5 ADCC correlates with S2M2-30-specific antibody protection

**Figure 5:**
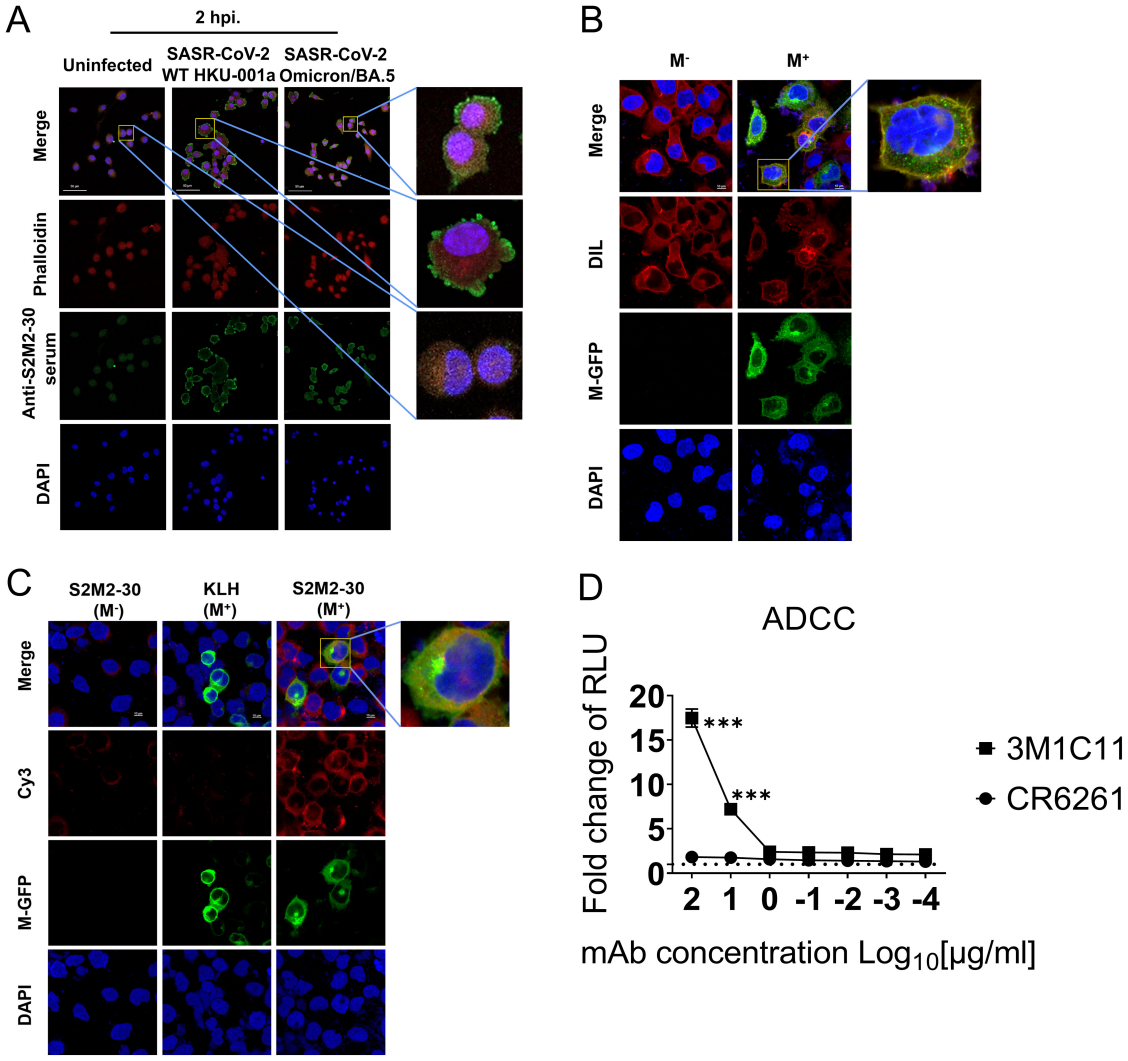
ADCC reporter assay. **(A)** Immunostaining of SARS-CoV-2-infected A549-TMPRSS2-ACE2 cells, using S2M2-30-specific hyperimmune serum. The uninfected cell culture was used as the negative control in immunostaining analysis. Phalloidin-iFluor dye (red) and DAPI (blue) were used for staining F-actin and nucleus in cells, respectively. A fluorescently labeled secondary antibody, Alexa Fluor 488 Goat Anti-Mouse IgG H&L (green), was utilized for signal amplification in the detection of the primary anti-S2M2-30 mouse serum antibodies. **(B)** Confocal fluorescence microscopy analysis of the transfected HEK293T cells overexpressing the recombinant M-GFP fusion protein (green) composed of the M protein and GFP. DIL dye (red) and DAPI (blue) were used for staining cellular membranes and nuclei in cells, respectively. **(C)** Confocal fluorescence microscopy analysis of the S2M2-30-specific serum-immunostained HEK293T cells overexpressing the M-GFP fusion protein (green). Fluorescently labeled secondary antibody Cy3 Goat Anti-Mouse IgG H&L (red) was utilized for signal amplification of primary mouse antibody detection (anti-S2M2-30 or anti-KLH serum). **(D)** ADCC surrogate assay with mAbs and transiently transfected HEK293T target cells overexpressing M protein (n = 2). An influenza hemagglutinin-specific mAb CR6261 was used as negative control. RLU, Relative light units. Data are presented as mean ± SEM. Multiple *t*-test were performed to statistically analyze the significance between the Preimmune group and S2M2-30-KLH group, as well as between the mAb CR6261 group and mAb 3M1C11 group. The *** represents *P* values less than 0.001.

ADCC activity contributes substantially to protection against SARS-CoV-2 infection (28–30). In this study, we investigated the correlation between S2M2-30-specific serum and ADCC activity. Immunofluorescence staining experiments confirmed the presence of numerous M proteins on the plasma membrane of A549-ACE2-TMPRSS2 cells infected with HKU-001a and Omicron/BA.5, as well as HEK293T cells transfected with a recombinant plasmid expressing M-GFP fusion protein (**Figures 5A–C**). To test ADCC activity *in vitro*, an S2M2-30-specific mAb, named 3M1C11, was isolated from S2M2-30-KLH-immunized BALB/c mice, by utilizing single B cell sorting-based amplification. Amino acid sequences of the heavy and light chains of 3M1C11 are listed in **Supplementary Table S2**. Sodium dodecyl sulfate polyacrylamide gel electrophoresis (SDS-PAGE) and Coomassie blue staining showed high purity of prepared mAb (**Supplementary Figure S5A**). ELISA results showed strong binding activity of 3M1C11 to S2M2-30 peptide (**Supplementary Figure S5B**). The Jurkat–Lucia NFAT-CD16 cells and the transfected HEK293T cells expressing full-length M protein were used as the effector cells and target cells, respectively. The 3M1C11 effectively elicited ADCC activity against the transfected HEK293T cells expressing M protein *in vitro* (**Figure 4D**).

## 4 Discussion

SARS-CoV-2 variants can cause breakthrough infections and lead to attenuated protection provided by vaccination through the mechanisms that escape antibody neutralization (31, 32). This study focuses on the conserved ectodomains of coronavirus M proteins to explore an additional mechanism of antibody protection against SARS-CoV-2. We demonstrate that S2M2-30-specific antibodies can be induced by SARS-CoV-2 infection and a booster dose of inactivated SARS-CoV-2 vaccine as well. Mouse immunization experiment results indicate that the S2M2-30-based vaccine confers protection through the elicitation of serum neutralizing activity (**Figures 3F, G**, **4D–F**), ADCC activity (**Figure 5**), and presumably the specific T-cell response (**Figures 3D, E**). Of note, the successful isolation of an ADCC-inducing S2M2-30-specific mAb confirms a novel protection mechanism for M protein-specific immunity (**Figure 5D**, **Supplementary Figure S5**). Our findings suggest that the presence of M protein ectodomain-specific immunity in the population should function protectively.

The antibody responses to the N-terminal peptides of SARS-CoV-2 M protein were often detected in patients during both the acute and convalescent phases of COVID-19 (14, 15), which are largely consistent with our findings (**Figure 2A**, **Supplementary Figure S2**). Our study further reveals that inoculation with an inactivated virus vaccine can also elicit a specific antibody response targeting the ectodomain of the M protein, and the its magnitude is correlated with the number of vaccinations. A complete B-cell epitope has been identified within the S2M2-30 peptide, while those truncated peptides do not have B-cell epitopes (**Supplementary Figure S3**). It was previously argued that the S2M21-30 peptide is located within the trans-membrane region (**Figure 1A**) (9). However, our findings suggest that this fragment is most likely not embedded in the viral envelope but fully exposed for B-cell recognition, as evidenced by that the fragment 21 - 30 is indispensable for S2M2-30 to induce high-affinity IgG response (**Supplementary Figure S3A**). Moreover, the compact architecture of the tandemly arranged viral M proteins observed using cryo-electron microscopy also indicates a restricted inter-protomer space for accommodating a lipid layer (9). The presence of quaternary epitopes on the M protein ectodomain remains unclear at present.

The neutralizing activities of S2M2-30-specific serum were observed in microneutralization assays (**Figures 3F, G**, **4D–F**). The possible mechanism through which S2M2-30-specific antibodies neutralize SARS-CoV-2 arouses our curiosity. A tomogram of the SARS-CoV-2 virion showed that the length distribution of nearest-neighbor distances between S proteins on the virion surface is approximately 23.6 nm (33). We speculate that this spacing is adequate for extensive antibody bindings to M protein ectodomains beneath S proteins on the virion surface (**Figure 5A**). These bound antibodies may potentially interfere with post-fusion structural changes in S proteins by steric hindrance (33, 34). It has been demonstrated that CR9114 antibody can sterically inhibit the activities of influenza neuraminidase upon binding to viral hemagglutinin (35, 36), indicating that the binding of a specific antibody to one viral surface antigen may inhibit the biological activity of another co-displayed antigen. Assembled SARS-CoV-2 virions bud into the intracellular compartment endoplasmic reticulum-Golgi intermediate compartment (ERGIC) lumen and are released into the extracellular space after the membrane fusion between virus-containing vesicles and plasma membrane (33, 37). Therefore, the presence of S2M2-30-specific neutralizing antibodies may not directly impede viral uncoating and release.

It has been reported that the S-specific serum antibodies isolated from COVID-19 patients who had been infected for several months are still capable of inducing ADCC activity, which may contribute to long-term immune protection (28–30). The *in vitro* experimental results of this study suggest that ADCC could potentially function as a protective mechanism for S2M2-30-specific immunity (**Figure 5D**), thereby also confirming the distribution of M proteins on the plasma membrane (**Figures 5A–C**). M proteins participate in the membrane curvature process and interact with other viral structural proteins, such as the envelope protein and S protein, in the intracellular vesicle structures to assist viral budding (38–41). We speculate that the unincorporated M proteins in the membrane of vesicles carrying virion particles may be delivered to and presented on the plasma membrane after membrane fusion and virus release. It is important to note that current research on the effector function of M protein-specific antibodies mainly relies on the use of recombinant M proteins in *in vitro* experiments. Consequently, the findings may not accurately reflect the actual mechanism of M protein-specific antibodies for *in vivo* protection. Further investigations are still necessary to elucidate their protection mechanism. Based on the findings of current research, we propose that the primary *in vivo* antiviral mechanism of M protein ectodomain-specific antibodies definitely involve both viral neutralization and ADCC activity.

Our findings also suggest that S2M2-30 peptide-specific T cell immune response may contribute to the *in vivo* immune protection. The restimulation of lymphocytes from spleen with S2M2-30 peptide significantly increased the numbers of interferon (IFN)-γ secreting lymphocytes and interleukin (IL)-4 secreting lymphocytes (**Figures 3D, E**). IFN-γ is a typical cytokine produced by Type 1 T helper (Th1) cells and exert antiviral effects primarily through its pleiotropic roles in priming macrophage activations, inducing apoptosis in infected cells (42, 43), as well as promoting mouse IgG2a subclass switch. IL-4 is a major stimulus for the development of type 2 Th (Th2) cells; it not only stimulates the expression of IgG1 but also drives proliferation of B cells along with other Th2-associated cytokines (44, 45). Moreover, T cell responses targeting the conserved epitopes from S2M2-30 hold significant promise for conferring broad protection against SARS-CoV-2 variants.

Due to the substantial number of mutations present in viral spike proteins, numerous Omicron subvariants possess a pronounced ability to evade neutralizing immunity conferred by SARS-CoV-2 vaccines (46, 47). Likewise, our findings indicate that vaccinations with wild-type RBD resulted in a significant yet modest inhibition of *in vivo* replication of B.1.1.529/Omicron BA.1 in mouse models. However, S2M2-30 peptide-specific immunity demonstrated an inability to exert any inhibitory effect on the replication of the Omicron virus (**Supplementary Figure S4A**). As clearly shown in **Figure 3A**, the mutations D3G/N and Q19E, which are exclusive to the M protein of Omicron strains (**Figure 1C**) do not affect the binding capacity of S2M2-30-specific antibodies. Given the inferior pathogenicity exhibited by available Omicron strains in our laboratories when assessed using the K18-hACE2 transgenic mouse model, we used an Omicron BA.1 virus with a PFU tenfold greater than that of B.1.1.7/Alpha (UK) for mouse infection (**Supplementary Figures S1B, C**). Presumably, this much larger quantity of B.1.1.529/Omicron BA.1 virus within inocula cause the diminished efficacy observed with S2M2-30-KLH vaccines (**Supplementary Figures S4B, C**).

The ectodomain of M protein has the potential to induce protective antibodies, as indicated by results from mouse immunization experiments demonstrating *in vitro* and *in vivo* cross-protection elicited by antibody responses targeting this domain. Based on the antibody protection mechanisms revealed in this study, it can be speculated that these specific antibodies also possess antiviral functions in humans. However, conducting a reliable retrospective cohort analysis to determine the impacts of the M protein antigen in inactivated vaccines on the induction of immune responses specific to vaccine components and their protectiveness is challenging. It is not entirely rigorous to analyze the roles of M protein by comparing immune responses elicited in the cohorts vaccinated with inactivated vaccines, mRNA vaccines, and recombinant protein vaccines. Additionally, we found that S2M2-30-KLH exhibited an *in vivo* antiviral efficacy closer to that of the RBD group compared to results from *in vitro* serum neutralization assays (**Figures 4C, D**), suggesting that specific cellular immunity contributes to its antiviral effect. Overall, the findings in this study deepen our understanding of the immunology related to M protein and provide valuable insights for developing broadly protective SARS-CoV-2 vaccines.

## Data Availability

The original contributions presented in the study are included in the article/**Supplementary Materials**, further inquiries can be directed to the corresponding author/s.
