# Supplementary material for: M protein ectodomain-specific immunity restrains SARS-CoV-2 variants replication
Source: Front Immunol. 2024 Oct 2;15:1450114. doi: 10.3389/fimmu.2024.1450114 (PMC11480003; doi:10.3389/fimmu.2024.1450114)
Supplement: Supplementary file 1 [file DataSheet1.docx]

Supplementary Material

# Supplementary Figures and Tables

## Supplementary Figures

**Supplementary Figure 1.** Mice immunization and infection experiment schedules. (A) The BALB/c mice received triple intraperitoneal immunizations with homotypic vaccines, including peptide-KLH conjugates emulsified with IFA, KLH emulsified with IFA, or RBD-hFc emulsified with IFA. (B, C) The K18-hACE2 mice received sequential intraperitoneal immunizations with 2-dose of either RBD-hFc emulsified with IFA or PBS, followed by 3-dose of either peptide-KLH conjugates emulsified with IFA, KLH emulsified with IFA, or PBS. After the last vaccination, the K18-hACE2 mice were intranasally infected with (B) 10^4^ PFU of B.1.1.7/Alpha (UK) or (C) 10^5^ PFU of B.1.1.529/Omicron BA.1. The clip art images from this figure are derived from the open-source website <https://openclipart.org/>.

**Supplementary Figure 2.** M protein ectodomain-specific IgG antibody responses in the study population. AUC for individual sera against M protein ectodomain peptides are shown. Serum samples were collected from convalescent COVID-19 patients (n = 10), individuals immunized with 2 doses (n=10) of CoronaVac inactivated vaccine, and outpatient children in mid-2019 (n = 10). All data are represented as mean ± SEM. Dashed lines indicate the mean + 2 × SD of the group of serum collected in mid-2019. Statistical significance between compared groups was determined using an unpaired *t*-test. *P* values less than 0.05 are considered statistically significant and the ‘ns’ means not significant.

**Supplementary Figure 3.** Serological analysis of immunized BALB/c mice. (A) Titration of serum IgG specific to M protein ectodomain peptides and RBD protein. (B) Comparisons of specific IgG endpoint titers following the second boost immunization. (C - D) Specific IgG1 and IgG2a endpoint titers and IgG1/IgG2a ratio. (E) Specific IgM endpoint titers. All data are presented as mean ± SEM. The dashed lines represent the mean + 2 × SEM of the negative control group. Statistical significance between compared groups was determined using an unpaired *t*-test for panels A and B while a multiple *t*-test was used for comparing the IgG 1 with IgG 2a. *P* values less than 0.05 are considered statistically significant. The *, **, and **** represent *P* values less than 0.05, 0.01, and 0.0001, respectively, and the ‘ns’ indicates not significant.

**Supplementary Figure 4.** Evaluations of *in vivo* protection against B.1.1.529/Omicron BA.1 strain. (A) Analysis of the copy numbers of the B.1.1.529/Omicron BA.1 viral RdRp gene from mice nasal irrigation samples using reverse transcription qPCR. The nasal wash samples from each group were collected on the 4^th^ day post intranasal infection with 10^5^ PFU of B.1.1.529/Omicron BA.1. The copy numbers of all viral RdRp genes were normalized to β-actin housekeeping gene expression. (B) Body weight changes and (C) survival rates of the immunized mice were recorded for 14 days after the intranasal infection with 10^5^ PFU of B.1.1.529/Omicron BA.1. All data are presented as mean ± SEM. Statistical significance was determined with an unpaired *t*-test for panels A and B. *P* values less than 0.05 were regarded as statistically significant. The ‘ns’ indicates not significant.

**Supplementary Figure 5.** Characterization of S2M2-30-specific mAb 3M1C11. (A) SDS-PAGE and Coomassie blue staining analysis. The loaded samples are as follows, 1: BSA; 2: reduced 3M1C11 mAb sample; 3: unreduced 3M1C11 mAb sample. (B) The binding activity of mAb 3M1C11 towards S2M2-30 was analyzed using ELISA. Coating immunoplate with either BSA solution or PBS solution serves as a negative control in ELISA. All data are presented as mean ± SEM. Multiple *t*-test was performed to statistically analyze the significance between mAb 3M1C11 and PBS. The *, **, and **** represent *P* values less than 0.05, 0.01, and 0.0001.

## Supplementary Tables

**Supplementary Table 1.** K18-hACE2 mice immunization regimen.

| Groups | 1^st^ immunization | 2^nd^ immunization | 3^rd^ immunization | 4^th^ immunization | 5^th^ immunization | Interval (weeks) |
| --- | --- | --- | --- | --- | --- | --- |
| RBD/S2M2-30-KLH | RBD-hFc | RBD-hFc | S2M2-30-KLH | S2M2-30-KLH | S2M2-30-KLH | 2 |
| RBD | RBD-hFc | RBD-hFc | PBS | PBS | PBS | 2 |
| S2M2-30-KLH | PBS | PBS | S2M2-30-KLH | S2M2-30-KLH | S2M2-30-KLH | 2 |
| KLH | PBS | PBS | KLH | KLH | KLH | 2 |

**Supplementary Table 2.** Amino acid sequences of the heavy chain and light chain of the S2M2-30-specfic monoclonal antibody 3M1C11.

| 3M1C11 mAb | Amino acid sequences |
| --- | --- |
| Heavy chain | (**The sequence of murine IgG heavy chain variable region**) MKCSWVIFFLMAVVTGVNSEVQLQQSGAEIVKPGASVKLSCTASGFYIKVTYMHWVKQRPQQGLEWIGRIDPANGDTKYDPKFQGKATITADTSSNTAYLQLSSLTSEDTAVYYCSRSAGSYFDCWGQGTTLTVSSASTKGPSVFPLAPSSKSTSGGTAALGCLVKDYFPEPVTVSWNSGALTSGVHTFPAVLQSSGLYSLSSVVTVPSSSLGTQTYICNVNHKPSNTKVDKKVEPKSCDKTHTCPPCPAPELLGGPSVFLFPPKPKDTLMISRTPEVTCVVVDVSHEDPEVKFNWYVDGVEVHNAKTKPREEQYNSTYRVVSVLTVLHQDWLNGKEYKCKVSNKALPAPIEKTISKAKGQPREPQVYTLPPSRDELTKNQVSLTCLVKGFYPSDIAVEWESNGQPENNYKTTPPVLDSDGSFFLYSKLTVDKSRWQQGNVFSCSVMHEALHNHYTQKSLSLSPGK (**The sequence of the constant region in human IgG1 heavy chain**) |
| Light chain | (**The sequence of murine IgG light chain variable region**) MDFQVQIFSFLLISASAIMSRGQIVLTQSPAIMSASLGERVTMTCTASSSVSSNYLHWYQQKPGSSPKLWIYRTSNLASGVPARFSGSGSGTSYSLTISSMEAEDAATYYCHQYHRFPWTFGGGTKLEMKRTVAAPSVFIFPPSDEQLKSGTASVVCLLNNFYPREAKVQWKVDNALQSGNSQESVTEQDSKDSTYSLSSTLTLSKADYEKHKVYACEVTHQGLSSPVTKSFNRGEC (**The sequence of the constant region in human IgG1 κ chain**) |
