# Supplementary figures and images for: M protein ectodomain-specific immunity restrains SARS-CoV-2 variants replication
Source: Front Immunol. 2024 Oct 2;15:1450114. doi: 10.3389/fimmu.2024.1450114 (PMC11480003; doi:10.3389/fimmu.2024.1450114)

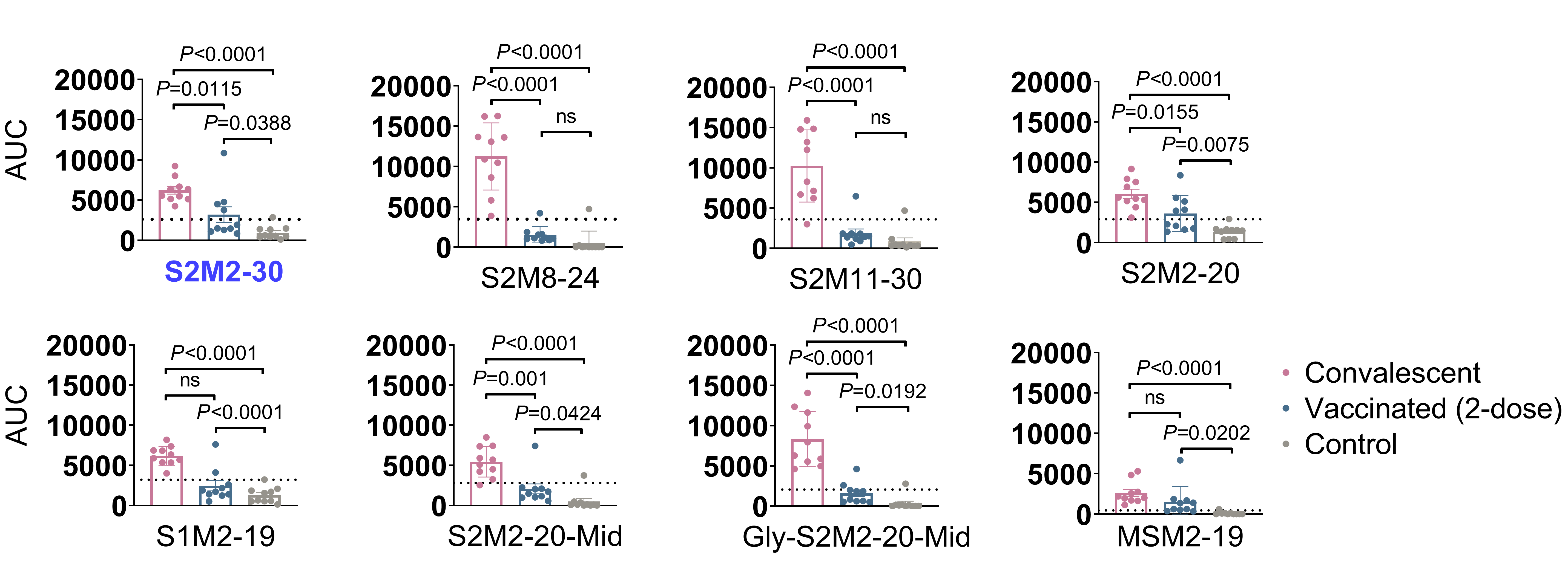

Supplement: Supplementary file 2 [file Image1.tif]

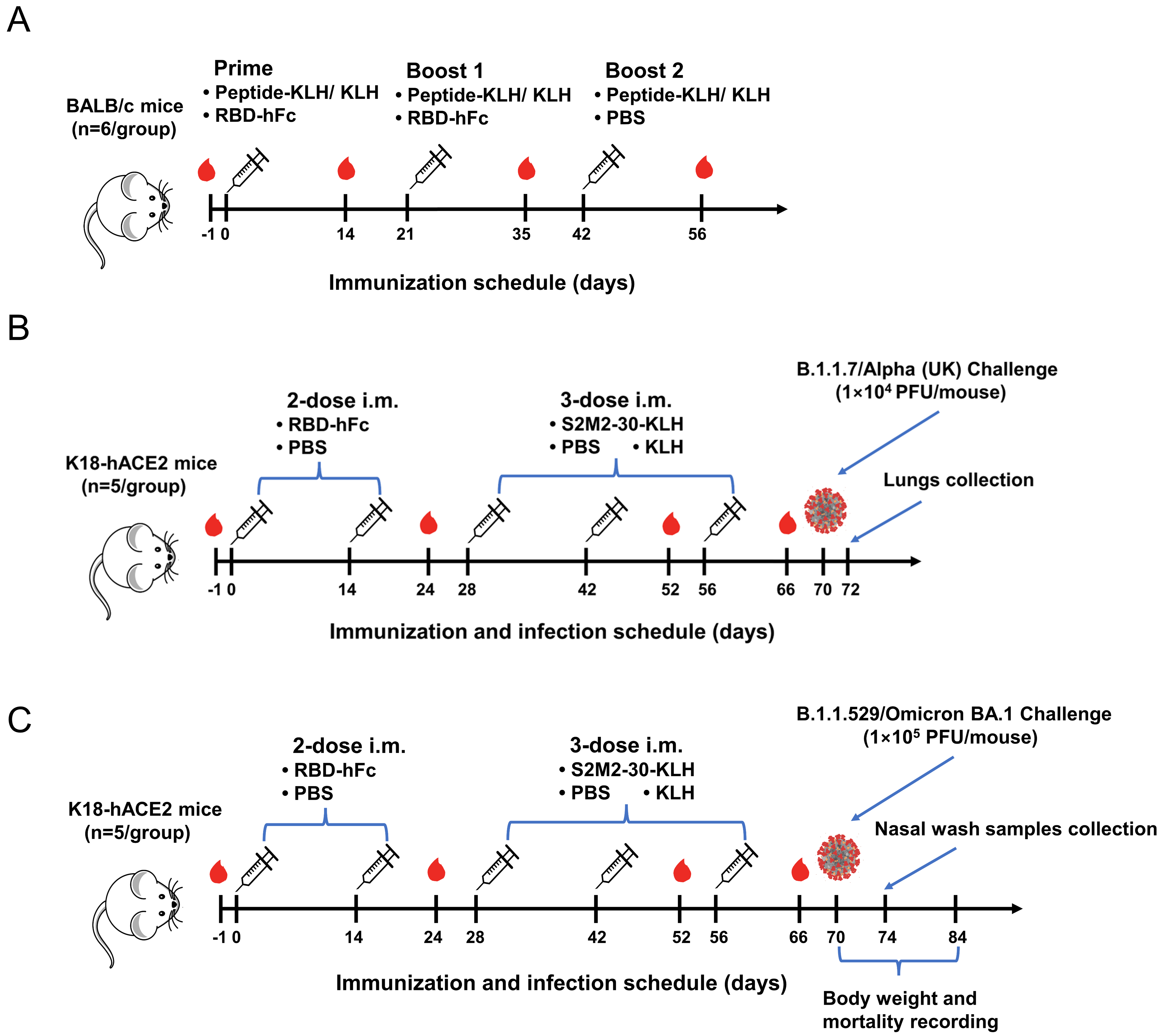

Supplement: Supplementary file 3 [file Image2.tif]

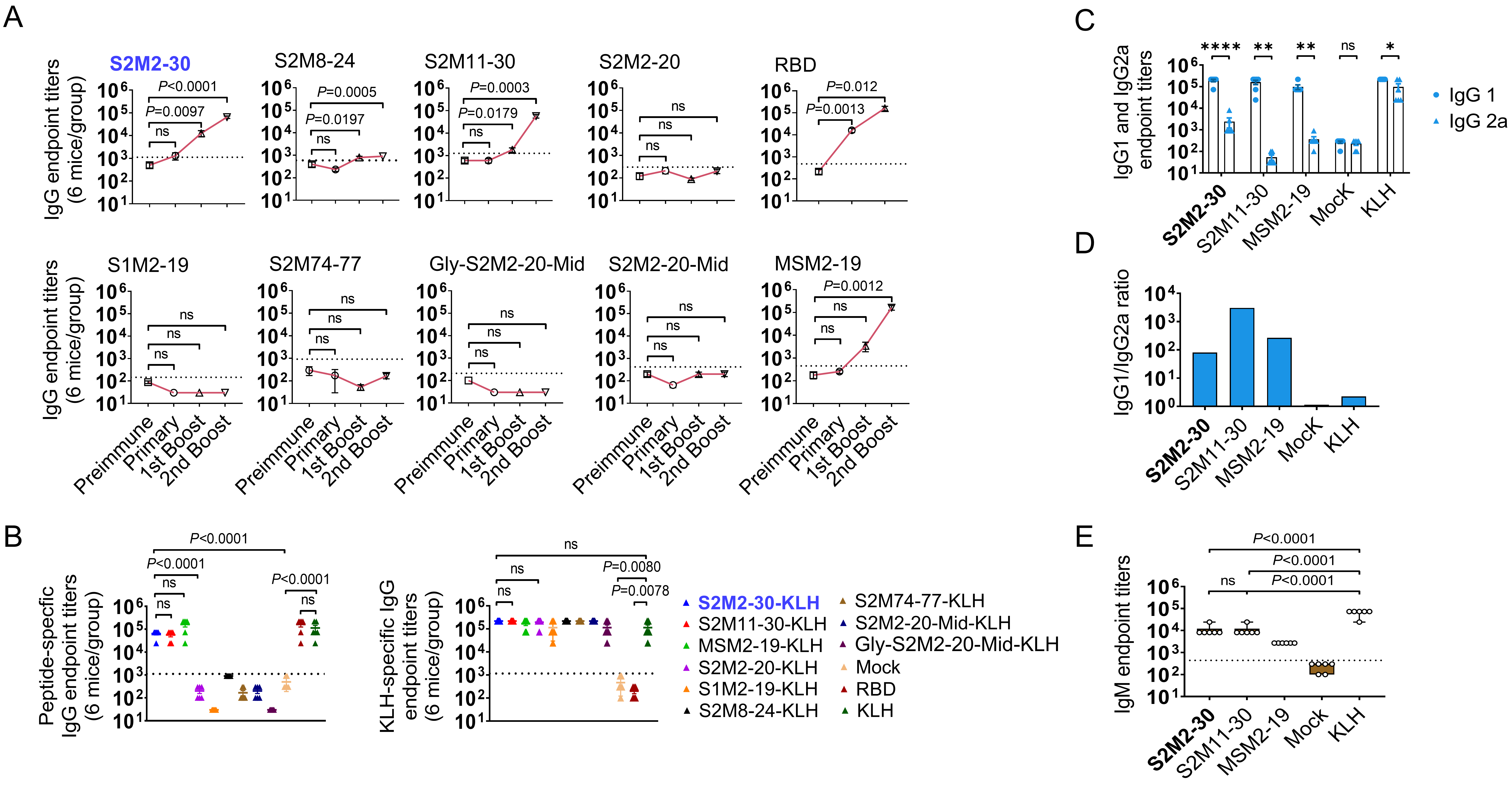

Supplement: Supplementary file 4 [file Image3.tif]

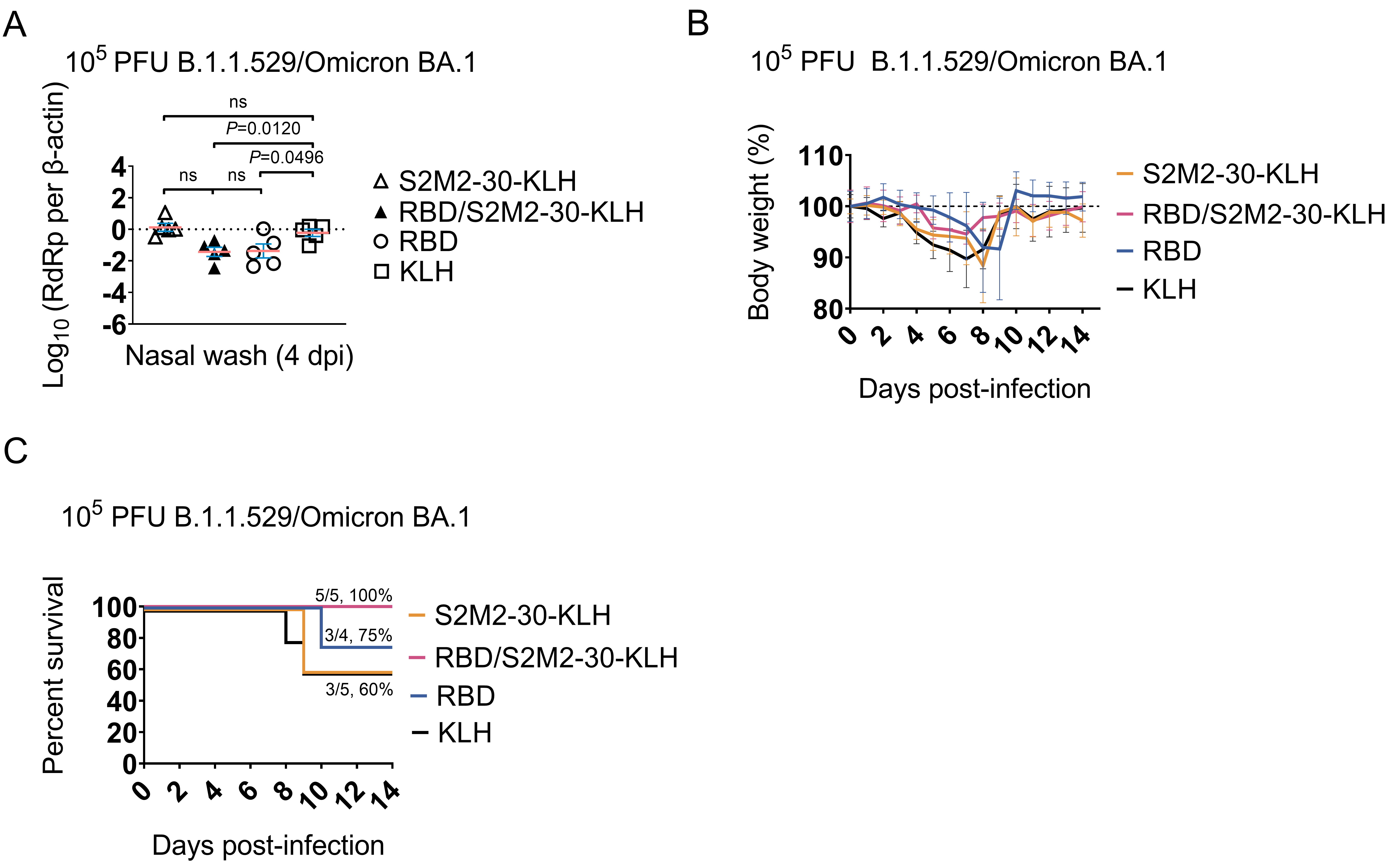

Supplement: Supplementary file 5 [file Image4.tif]

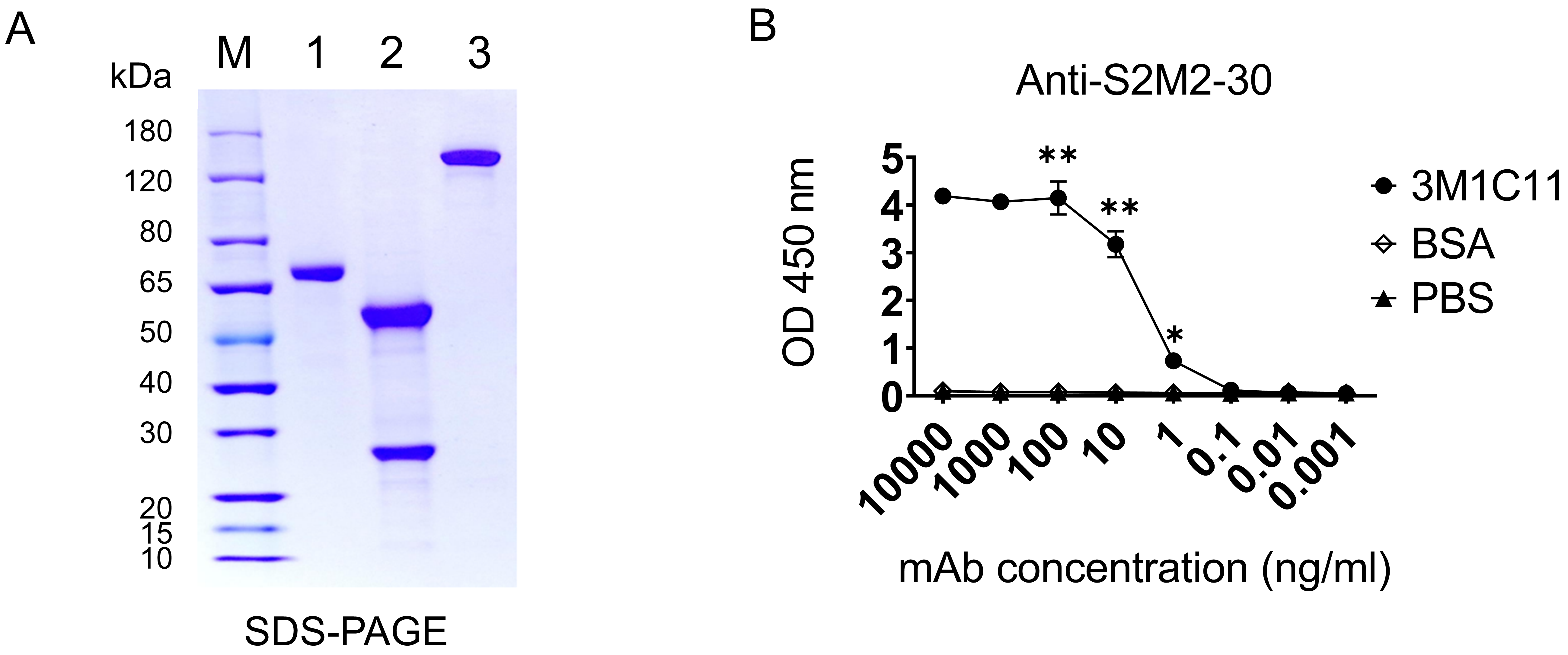

Supplement: Supplementary file 6 [file Image5.tif]
